# Modification of Paper Surface by *All*-Lignin Coating Formulations

**DOI:** 10.3390/ma15227869

**Published:** 2022-11-08

**Authors:** Patricia I. F. Pinto, Sandra Magina, Sara Fateixa, Paula C. R. Pinto, Falk Liebner, Dmitry V. Evtuguin

**Affiliations:** 1 RAIZ—Forest and Paper Research Institute, Quinta de S. Francisco, Apartado 15, 3801-501 Eixo Aveiro, Portugal; 2 CICECO—Aveiro Institute of Materials and Department of Chemistry, University of Aveiro, Campus Universitário de Santiago, 3810-193 Aveiro, Portugal; 3 Department of Chemistry, Institute of Chemistry of Renewable Resources, University of Natural Resources and Life Sciences, Vienna (BOKU), Konrad Lorenz Strasse 24, A-3430 Tulln, Austria

**Keywords:** cationic lignin, layer-by-layer coating, lignosulfonate, paper, free surface energy

## Abstract

*All*-lignin coating formulations were prepared while combining water-soluble cationic kraft lignin (quaternized LignoBoost^®^, CL) and anionic lignosulphonate (LS). The electrostatic attraction between positively charged CL and negatively charged LS led to the formation of insoluble self-organized macromolecule aggregates that align to films. The structures of the formed layers were evaluated by atomic force microscopy (AFM), firstly on glass lamina using dip-coating deposition and then on handsheets and industrial uncoated paper using roll-to-roll coating in a layer-by-layer mode. Coated samples were also characterized by optical microscopy, scanning electron microscopy (SEM) coupled with energy dispersive spectroscopy (SEM/EDS), and contact angle measurements. It was suggested that the structure of *all*-lignin aggregates is the result of the interaction of amphiphilic water-soluble lignin molecules leading to their specifically ordered mutual arrangement depending on the order and the mode of their application on the surface. The *all*-lignin coating of cellulosic fiber imparts lower air permeability and lower free surface energy to paper, mainly due to a decrease in surface polarity, thus promoting the paper’s hydrophobic properties. Moderate loading of lignin coating formulations (5–6 g m^−2^) did not affect the mechanical strength of the paper.

## 1. Introduction

Environmental concerns and the depletion of fossil fuels are motivating consumers to seek sustainable alternatives to plastic materials in packaging. Paper and paperboards are the most common packaging materials constituted of cellulosic fiber derived from plant resources. Being renewable, biodegradable, and with satisfactory viscoelastic properties, paper products are positioned as a versatile and sustainable alternative to plastics in packaging applications [1,2]. However, the significant loss in strength properties under moistening and poor barrier properties are limiting factors of paper materials [3]. The aforementioned limitations are due to the hygroscopic nature of cellulose and the high porosity of the cellulose fiber networks [4]. Usually, to overcome these drawbacks, paper or paperboard are surface-coated with waxes, combining organic–inorganic coating formulations or synthetic polymer films [5]. Even though hydrophobization of paper materials can be achieved by single-layer coating (in many cases at the expense of compromised mechanical and chemical properties), multiple layers are known to offer a range of opportunities with regard to additional functions and better fitting properties for a variety of packaging applications [6].

Sustainable and renewable materials such as natural polymers have been widely used as paper surface coating agents that include polysaccharides such as starch, chitosan, alginate, and fibrillated cellulose [7,8,9,10,11,12,13,14,15]. Recently, technical lignins have also shown promise with regard to improved barrier properties and moisture resistance. As a substantial part of coating formulations, they could additionally impart antioxidant, antimicrobial, or UV-blocking properties to paper-based packaging materials [16].

Although nowadays technical lignins are available as secondary streams from wood pulp and some biorefinery industries, they are mainly used for energy recovery and only 2% of them are used in materials and chemical applications [17]. Kraft pulping of wood carried out under strongly alkaline conditions is nowadays the dominant technology in pulp and paper production worldwide. The respective technical lignin, hitherto largely underutilized due to its poor water solubility (at pH < 9), is commonly referred to as kraft lignin. Sulfite pulping, which relies on the bond-cleaving capabilities of aqueous solutions of sulfur dioxide and an appropriate base, is another industrial process that is mainly used for the production of dissolving and special cellulosic pulps. The technical lignins recovered from this process are known as calcium, magnesium, ammonium, or sodium lignosulphonates, depending on the nature of the base used. Even though their importance has been receding, there are still over 10 million tons of lignosulphonates commercially available [16,17]. In contrast to kraft lignin, lignosulphonates are soluble in water across the entire pH scale due to the abundant sulfonic acid groups added to their structure during pulping. This feature of good water solubility is employed in a wide range of applications, where lignosulphonates act as dispersants, emulsifiers, adhesives, setting retarders, or feedstock to produce valuable chemicals [18]. As a consequence of the shifting dominance towards kraft technology, several approaches have been proposed to improve the solubility of kraft lignins in water significantly. This can be accomplished through the introduction of either sulfonic groups (anionic kraft lignin) [18] or quaternary ammonium groups (cationic kraft lignin) [19]. Both anionic and cationic kraft lignins as well as lignosulphonates are promising candidates for the modification of paper materials. Among reported paper coating approaches, lignins are mostly combined with inorganic particles/fillers [13,20] or with different polysaccharide blends [8,9,15,21] to substitute synthetic binders. Much less attention was paid to paper coating using lignins only, although it was demonstrated that the modification of cellulosic fibers combining cationic synthetic polymers with anionic-modified lignosulphonate in layer-by-layer assembly can provide quite hydrophobic surfaces and promising properties to papers produced from these modified fibers [14].

In this study, for the first time, the effect of paper coating using *all*-lignin coating formulations was examined using both water-soluble cationic and anionic lignins. Cationic kraft lignin was prepared by introducing quaternary ammonium groups and anionic lignin was a purified lignosulfonate. When combined with each other, these two lignins are able to form a film that is insoluble both in water and in organic solvents. The structures of the *all*-lignin films were primarily evaluated by atomic force microscopy (AFM) after the dip-coating of glass lamina and later on paper. Aiming to assess the strength and surface properties of laboratory and industrial papers, respective specimens were coated with cationic/anionic formulations of the water-soluble lignin derivatives applied layer-by-layer using the roll-to-roll coating technique.

## 2. Materials and Methods

### 2.1. Reagents

*Eucalyptus globulus* LignoBoost^®^ kraft lignin was kindly provided by The Navigator Company (Aveiro, Portugal) and characterized in detail by Vieira et al. [22]. *Eucalyptus globulus* lignosulphonates (LS) were kindly supplied by Caima Company (Constância, Portugal) and characterized by Magina et al. [23]. Handsheets (HS) and base paper (paper) produced from bleached eucalyptus kraft pulp (BEKP) were supplied by The Navigator Company (Aveiro, Portugal). Both HS and paper were used without any surface treatment and had a basis weight of 65 and 80 g m^−2^, respectively. Sodium chloride, sodium hydroxide, and sulfuric acid were all ACS grade and supplied by Fisher Chemical (Loughborough, UK); hydrochloric acid (ACS grade) was obtained from Panreac (Darmstadt, Germany); 3-chloro-2-hydroxypropyl-trimethylammonium chloride (CHPTAC, 65 wt.% in H_2_O) was purchased from TCI Europe N.V. (Zwijndrecht, Belgium). Dialysis tubing (benzoylated cellulose, cut-off 2000 Da) was obtained from Sigma-Aldrich (Lisbon, Portugal). All chemicals were used as supplied and deionized distilled water (2.67 µS m^−1^) was used in all experimental work.

### 2.2. Surface Coatings and Characterization

**Scheme 1:**
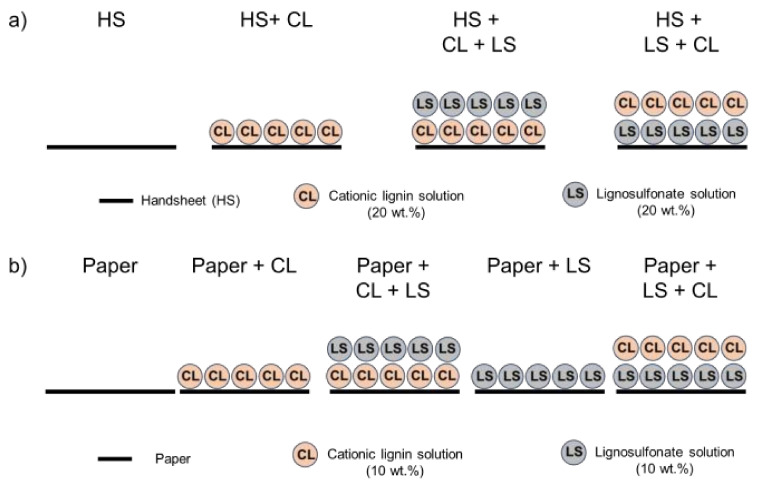
General design of coatings applied to different substrates: (**a**) handsheets (HS) with a concentration of 20 wt.% of cationic lignin (CL) and lignosulfonates (LS) and (**b**) papers with a concentration of 10 wt.% of CL and LS.

Handsheets (HS) and base paper (paper) were used as cellulosic substrates. These substrates were coated as represented in Scheme 1. All coatings were applied using a manual K lox proofer-RK Print Coater (RK PrintCoat Instruments Ltd., Litlington, UK). CL and LS were prepared in aqueous media at two concentrations, 20 wt.% and 10 wt.%, respectively, and applied one at each time and in a different order, to the HS and paper (cf. Scheme 1). Drying of each layer was performed on air and subsequently in a ventilated oven (40 °C, 6 h). The intermediate drying between coatings was carried out at 80 °C for 15 min. The coated samples were conditioned at 23 ± 1 °C and 50 ± 2 % RH for 24 h. Grammage, thickness, tensile strength, roughness, and air permeation (Bendtsen method) were measured on selected samples and according to ISO standards: ISO 536, ISO 534, ISO 1924-2, ISO 8791-2, and ISO 5636-3, respectively. According to ISO 5636-3, the paper air permeability is defined as the volume of air (mL/min) passing through the measuring head under defined pressure. The water drop test consisted of placing a drop of water (ca. 0.1 cm^3^) on the surface of the coated paper and observing the washout of the coating for 1 h at room temperature.

The coating of 22 mm × 22 mm and 170 μm thickness glass laminae (Cover glass, AuxiLab S.L., Berain, Navarra, Spain) with lignins was carried out using RDC 21-K Dip Coater (Bungard Electronik GmbH & Co. KG, Windeck, Germany) at a velocity of 200 mm min^−1^. The glass laminae were cleaned with ethanol and dried before use. After coating, excess lignin solution (CL or LS, 5 wt.%) was left to drain off for 5 min before removing remnants by absorption paper from the bottom. The intermediate coated layer was dried for 15 min at 80 °C in the ventilated oven in limbo before the second layer was applied. The final drying was accomplished in a ventilated oven at 40 °C for 6 h. The coating thickness varied between 500 and 800 nm. The coated glass laminae were kept in a closed glass box and stored in a desiccator in darkness at room temperature.

Contact angle measurements were performed on an OCA20 goniometer (Data Physics Instruments, Filderstadt, Germany) equipped with a CCD camera and SCA20 software. The sessile drop method was applied using water, formamide, and diiodomethane as probe liquid [24,25]. For each paper sample the contact angle was measured for a minimum of 6 micro-droplets (3 μL for water) deposited on different sites of the paper surface. The results were averaged and the standard deviations were evaluated. The surface energy ($\gamma_{s}$) of the paper surface including its corresponding polar ($\gamma_{s}^{p}$) and dispersive ($\gamma_{s}^{d}$) components was determined according to the Owens–Wendt–Rabel–Kaelble (OWRK) model [26,27]. The OWRK model is described in Equation (1):

(1) $$\frac{1+cos\theta }{2}\times \frac{\gamma_{l}}{\sqrt{\gamma_{l}^{d}}}=\sqrt{\gamma_{s}^{p}}\times \sqrt{\frac{\gamma_{l}^{p}}{\gamma_{l}^{d}}}+\sqrt{\gamma_{s}^{d}}$$

where $\gamma_{l}$, $\gamma_{l}^{p}$, and $\gamma_{l}^{d}$ represent the liquids’ superficial tension and the corresponding polar and dispersive components, respectively. Plotting $\left(1+cos\theta \right)/2$ vs. ${\left(\gamma_{l}^{p}/\gamma_{l}^{d}\right)}^{1/2}$ allows the calculation of the surface polar and dispersive parameters $\gamma_{s}^{d}$ and $\gamma_{s}^{p}$ [24].

It is worth mentioning that the linear regression parameters were determined using all measured data (a minimum of six readings of the contact angles for each liquid probe) instead of using the averaged readings for each liquid. Consequently, the confidence intervals are statistically significant.

Optical microscopy images were acquired in reflectance mode and recorded on a Hi-rox Rh-2000 instrument (Olympus Corp., Tokyo, Japan) equipped with an MXB-2500Rez camera. SEM images of handsheet surfaces after gold sputtering were recorded using a Hitachi S-4100 microscope (Hitachi, Tokyo, Japan), at an acceleration voltage of 5 kV. SEM/EDS images were taken using a TM4000Plus tabletop SEM microscope (Hitachi, Tokyo, Japan) operated with an accelerating voltage of 15 kV and equipped with an EDS QUANTAX 75 detector (Bruker Corporation, Billerica, MA, USA).

Atomic force microscopy (AFM) analysis of coated with lignin glass laminae and papers was carried out in tapping mode on a Confocal Raman-AFM-SNOM WITec alpha 300RAS^+^ instrument (WITec, Ulm, Germany), using a silicon tip (NC tips) and cantilever, the latter equipped with reflex-coating on the detector side. A spring constant of k = 42 N m^−1^ and a resonance frequency of 285 kHz were used. The scanning areas were 50 μm × 50 μm and 20 μm × 20 μm (512 points per line × 512 lines per image) scanned (and retraced) at a speed of 2 s/line, and 5 μm × 5 μm (256 points per line × 256 lines per image) scanned (and retraced) at a rate of 1 s/line. The surface roughness and arithmetic mean height (*S_A_*) were evaluated using the software Project 5.3^+^.

## 3. Results and Discussion

Previously well-characterized cationic lignin (CL) derived from LignoBoost^®^ eucalyptus kraft lignin [19] and anionic purified lignosulphonate (LS) from acidic magnesium sulfite pulping of eucalyptus wood [23] were used in this study for the *all*-lignin paper coating. A representative cationized lignin sample was selected (QL-5) since previously it demonstrated its excellent water solubility independent of the pH (test range pH 2–12) [19]. Although CL had lower molecular weight than LS (ca. 2.2 against 4.1 kDa) and a higher degree of substitution (DS) with quaternary ammonium groups than LS with sulfonic acid groups (DS of ca. 1.2 in CL versus ca. 0.6 in LS), the absolute zeta potentials of these two lignins were quite similar (+22.4 mV for CL and −21.3 mV for LS). It was verified that even at low solute concentrations (1 wt.%) mixing of CL and LS results in the precipitation of a solid insoluble in water and common organic solvents (e.g., acetone or ethanol). The maximum amount of precipitate was obtained when approximately equal volumes of the two solutions were combined (Figure S1, Supplementary Materials). The formation of this precipitate is the result of coulombic (electrostatic) attraction between the positively charged quaternary ammonium groups abundant in CL and the negatively charged sulfonate moieties of LS leading to the formation of self-organized macromolecular aggregates. Such solvent-resistant aggregates, and in particular their hydrophobicity, can be of great benefit for bio-based *all*-lignin coating formulations. Aiming to investigate the structure of the CL/LS films, the latter were formed on the surface of glass lamina by dip-coating. Additionally, laboratory-made handsheets and industrial papers were coated using a common roll-to-roll technique.

### 3.1. Surface Coatings on Glass Lamina

Thin (ca. 80 μm) glass laminae possessing plane smooth homogeneous negatively charged surfaces were selected to study in situ the self-organized structures of the *all*-lignin films, simulating to some extent those formed on the paper surfaces. Deposition of the films on the glass slides was carried out in two modes: (i) with intermediate drying of the deposited lignin layer or (ii) without drying and onward processing in a wet state. For both approaches, the surfaces of the obtained layers were evaluated for their morphology by AFM and water contact angles.

**Figure 1:**
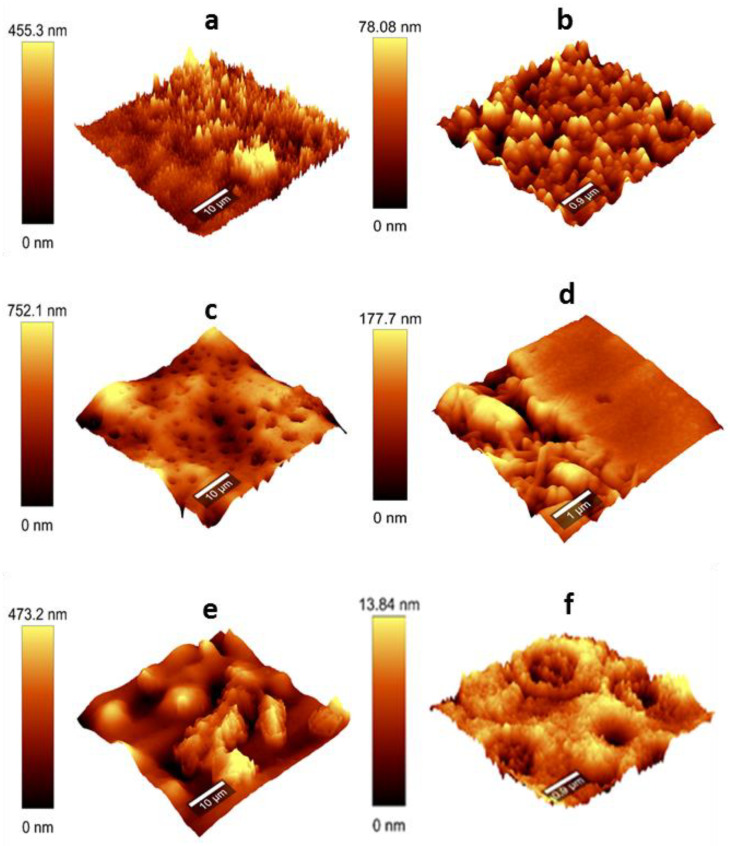
Images of 3D AFM surface topology of the glass lamina coated with CL and LS (CL + LS) (**a**,**b**) and with LS and CL (LS + CL) (**c**,**d**) with intermediate drying of deposited layers and with CL and LS (CL + LS) (**e**,**f**) without intermediate drying of deposited layers. The size of images varied from 5 µm × 5 μm (**b**,**d**,**f**) to 50 µm × 50 μm (**a**,**c**,**e**).

**Figure 2:**
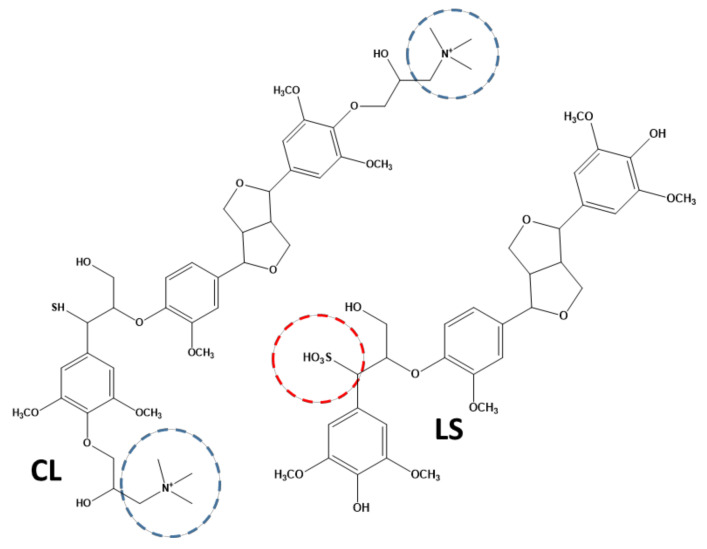
Schematic representation of cationic eucalyptus lignin (CL) and eucalyptus lignosulphonates (LS). The quaternary ammonia and sulfonic groups outlined by blue and red circles, respectively.

It was verified that the deposition mode plays an important role in macromolecular self-organization and, hence, the structure of the *all*-lignin films (ALFs) formed. Thus, the consecutive deposition of CL and LS (CL + LS) or of LS and CL (LS + CL) with intermediate drying of deposited layers, resulted in surfaces featuring pronounced bulges (CL + LS) or deep depressions (LS + CL) (Figure 1a,c). Interestingly, the surface roughness of the layers formed by the two opposing variants of intermediate drying was very similar, i.e., within the arithmetic mean height (*S_A_*) range of 40–80 nm. In contrast, the topology of CL + LS films produced without intermediate drying of the deposited layers was considerably smoother due to the much shallower and broader bulges and valleys formed (Figure 1), giving rise to a somewhat lower surface roughness of 30–60 nm (*S_A_*). A more detailed analysis of the valleys (image 5 μm × 5 μm, Figure 1f) showed that they were composed of conjugated alternating donut-like aggregates, some of which were empty, whereas others were filled with embedded concentric structures. Such donut-like aggregates are typical for amphiphilic macromolecules possessing both polar (“head”) and non-polar (“tail”) counterparts [28,29]. In the case of CL and LS, these polar counterparts consist of structural units carrying quaternary ammonium (CL) or sulfonic acid (LS) groups (Figure 2), whereas the respective phenyl propane structural fragments represent the much less polar fraction of the lignins.

The self-organization potential of amphiphilic compounds depends on many factors, such as the molecular geometry (e.g., molecule shape and hydrodynamic radius, chain flexibility, steric hindrance, intra and intermolecular bonds) and the solution conditions (concentration, temperature, pH and ionic strength). It can be characterized by the critical packing factor (*C_PP_*, Equation (2)), which is defined as the ratio between the effective volume occupied by hydrophobic chains in the aggregate core (*V*_0_) and the effective hydrophobic chain length (*L_C_*) and the effective hydrophilic head group surface area (*A_mic_*) [29]:

(2) $$C_{PP}=V_{\left(0\right)}/\left(A_{mic}*L_{C}\right)$$

**Figure 3:**
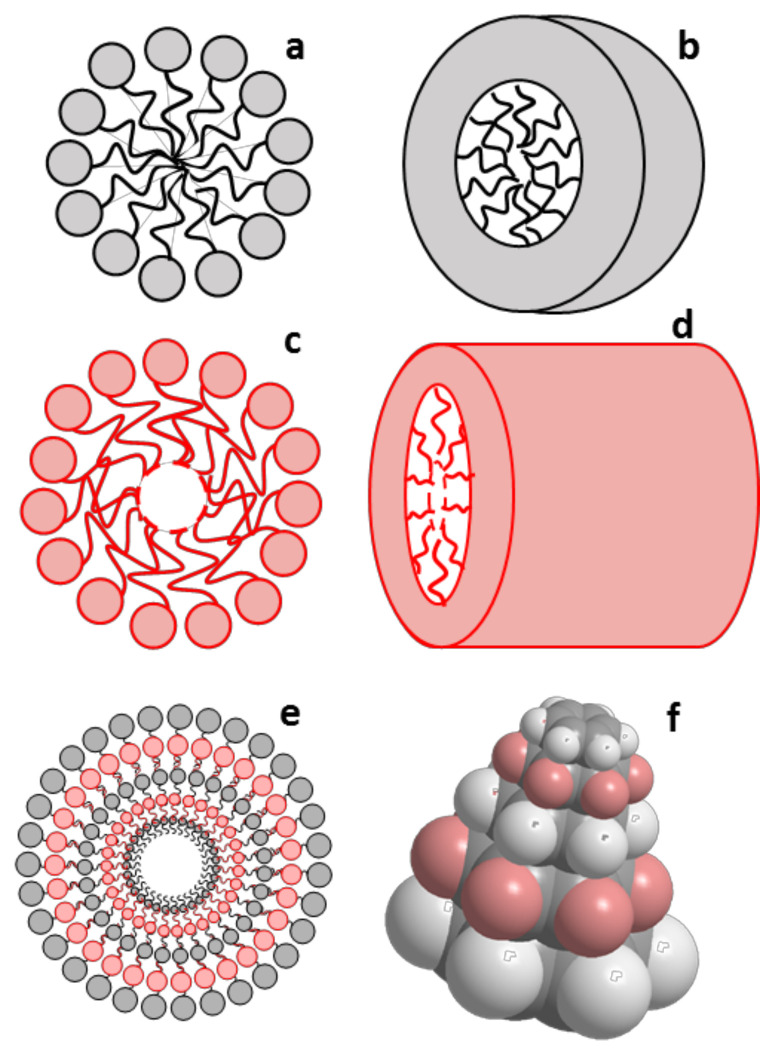
The 2D (**a**,**c**,**e**) and 3D (**b**,**d**,**f**) schematic representations of lignin aggregates shown as an assembly of amphiphiles with polar (balls) and non-polar (tail-like crooked lines) counterparts. Depending on the critical packing factor (*C_PP_*), the shape of the aggregate micelles can be spherical (**a**,**b**) or cylindrical (**c**,**d**). The positively charged polar counterparts of lignin are depicted by grey balls (in CL) and the negatively charged ones by pink balls (in LS). The interaction of positively and negatively charged lignins can form the observed cone-shaped aggregates (**e**,**f**).

If *C_PP_* is very low (≤1/3), the structure of aggregates can be spherical, at 1/3 < *C_PP_* < 1/2 a cylindrical shape is more likely, and lamellar structures are commonly found, when *C_PP_* is close to 1 [29]. In this study, the shape of most of the lignin aggregates present on the surface of LS + CL film (except for regions with depressions) resembles that of loosely connected small spheres (Figure 1d), whereas those on the surfaces of the CL + LS films appeared to be composed of rather jointed ellipsoid rods aligned perpendicular to the surface of the glass lamina (Figure 1b). This could be assigned to the higher *C_PP_* of LS aggregates surpassing that of the CL aggregates (Figure 3). It is assumed that this is caused by the higher molecular weight of LS, being twice as high as CL (i.e., of higher *V*_0_), and its lower degree of substitution with polar groups (sulphonate moieties) compared to CL (i.e., of lower effective *A_mic_*).

It is to assume that the extent of interaction between the negatively charged LS and positively charged CL layers depends on whether an intermediate drying of the deposited layers was granted or not. Thus, when depositing lignin from the solution state onto the previously dried layer, the interaction of oppositely charged aggregates is limited. Nevertheless, some mobility of both types of lignin aggregates might explain why the above-described elongated cone-shaped assemblies, supposedly composed of alternating oppositely charged lignin aggregates, were formed (Figure 3). The last assumption is supported by the characteristic bulge/depression surface topology of the CL + LS films (and to a lesser extent of the LS + CL samples), obtained with intermediate drying of deposited layers (Figure 1). In analogy to the leaf of the lotus flower (*nelumbo nucifera*), in particular, the surface of these cone-shaped formations on CL + LS films should be less susceptible to moistening and microbial infestation than other flatter regions constituted essentially of more homogeneous lignin aggregates (LS or CL).

The assembly of LS and CL lignins deposited without the intermediate drying of layers combines apparently different types of aggregates, the most common being donut-like assemblies of unknown configuration (Figure 1e,f). However, these propositions about the structure of the macromolecular aggregates formed require further in-depth studies. It is noteworthy that the films solely formed from CL or LS and deposited on top of the glass lamina featured irregular surface patterns of moderate roughness (*S_A_* = 40–70 nm) and a morphology similar to that observed in the outer layers of CL + LS or LS + CL films (Figure S2, Supporting Materials).

The water contact angles (WCA) of the different *all*-lignin films obtained in this work were dependent on both the type of lignin derivative and the way it was deposited on the glass lamina. Thus, the lowest WCA was observed for CL and the highest for the LS films (Table 1). This is in accordance with previous studies reporting relatively low water contact angles for films formed from cationic lignins [21,30]. However, in LS + CL composite films, where CL was the outermost layer at the film–air interface, WCA was higher than for the film composed of CL only (Table 1). This implies that the inner LS layer of the LS + CL composite film had a directing effect on the CL macromolecules. This is evident from the crater-rich surface formed by the cone-shaped lignin aggregates which also imparts lower hydrophilicity to LS + CL films (compared to neat CL films) as evident from their higher WCA (41.3 ± 1.0 vs. 32.5 ± 1.8). The occurrence of a similar directing effect for the CL + LS films was concluded from their peak-rich surface opposing that of LS + CL films. However, compared to neat LS films (WCA 68.4 ± 2.1) no further gain in hydrophobicity was observed for CL + LS films (WCA 61.7 ± 3.1). The relatively low affinity of CL + LS coatings towards water is paired with another great advantage that results from the specific order of lignin deposition for this variant: due to the negative electrical surface charges of glass lamina (as the paper surface), the initial deposition of CL allows for a more intimate bonding on the substrate surface than it would be the case when LS would form the first layer.

Indeed, besides electrostatic attraction, adhesion between the substrate surfaces and lignin might also involve Van der Waals and donor–acceptor interactions. The occurrence of these specific interactions was also evident in lignin-silica hybrids obtained by sol–gel synthesis [31]. Unlike CL, for which phenolic groups were etherified to carry positively charged 2-hydroxypropyl-3-trimethylammonium moieties, sulfonic and phenolic groups of LS can form hydrogen bonds with free hydroxyl groups on the surface of the glass lamina. This explains why lignin leaching (25 °C, 24 h) was less pronounced for LS than for CL coating. Furthermore, it is assumed that a higher fraction of hydrophobic counterparts of LS is exposed to the film–air interface, which could explain why the highest WCA was observed for the LS films (LS, Table 1). When the LS layer is deposited over the CL layer (CL + LS, Table 1), the WCA was lower than that of the film composed only of LS, indicating the effect of CL on the structure of the subsequent LS layer as discussed above. Presumably one of these effects can be the formation of cone-shaped bulges on the surface of CL + LS films (Figure 1a), which also contain the CL counterpart (Figure 3c), contributing to some decrease in WCA. The amount of these morphological elements decreases significantly when there is no intermediate drying between the deposition of the CL and LS layers ([CL + LS] assembly), thus slightly increasing the WCA (64.7 ± 2.8°) compared to the glass slides coated with the identical layer sequence but with implemented intermediary drying (61.7 ± 3.1, CL + LS; Table 1). For [CL + LS] assembly, a notable part of aggregates on the surface is composed of both CL and LS in the form of empty and filled “donuts” (Figure 1f) leading to some better affinity with water that it observed with films composed of solely LS (GL + LS, Table 1). Leaching in water (25 °C, 24 h) of the deposited films [CL + LS] and CL + LS was minimal, unlike the films composed only of CL or LS.

### 3.2. Surface Coating of Pulp Handsheets

In order to assess the coating of cellulosic fibers with *all*-lignin formulations, the latter were deposited first on the laboratory handsheets produced from bleached refined (30° SR) eucalyptus kraft pulp according to standard procedure (ISO 5269-2:2004). The laboratory handsheets (HS) are porous fiber mesh affected by the preparation mode. All depositions were carried out on the most visually homogeneous smooth part of the HS using a conventional roll-to-roll technique with intermediate drying of the deposited layers. In this case, the lignin formulations applied as aqueous solutions had the possibility of partial penetration inside the fiber network induced by capillary forces, thus not allowing the formation of a continuous film on the surface as in the case with the glass lamina.

**Figure 4:**
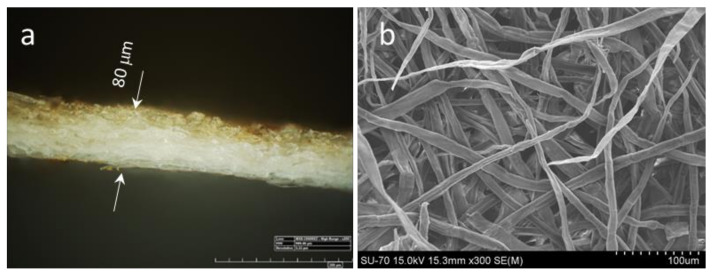
Cross-sectional image of the handsheet (HS) coated by CL and LS (CL + LS) obtained by optical microscopy (**a**) and the SEM image of the HS surface (**b**).

According to microscopy analysis of transversal cuts of coated HS, the penetration depth of lignin formulations is about 10–30 μm only at a lignin application load of ca. 4–5 g m^−2^ (Figure 4). Hence, the formulations reach the accessible fiber surface being capable to fill some micro- and mesopores but leaving the interfiber voids open. Nevertheless, the air permeability of the coated HS measured by the Bendtsen method (according to ISO 5636-3) decreased noticeably. Thus, the application of CL (ca. 3 g m^−2^) on the HS surface (HS + CL) led to a decrease in its air permeability from 70.7 cm^3^ min^−1^ to 58.9 cm^3^ min^−1^, which was very similar to values obtained when HS was coated at a similar load with LS (60.5 cm^3^ min^−1^). Subsequent deposition of a secondary lignin layer reduced the air permeability of the handsheets even further as demonstrated for variant HS + LS + CL (50.9 cm^3^ min^−1^).

**Figure 5:**
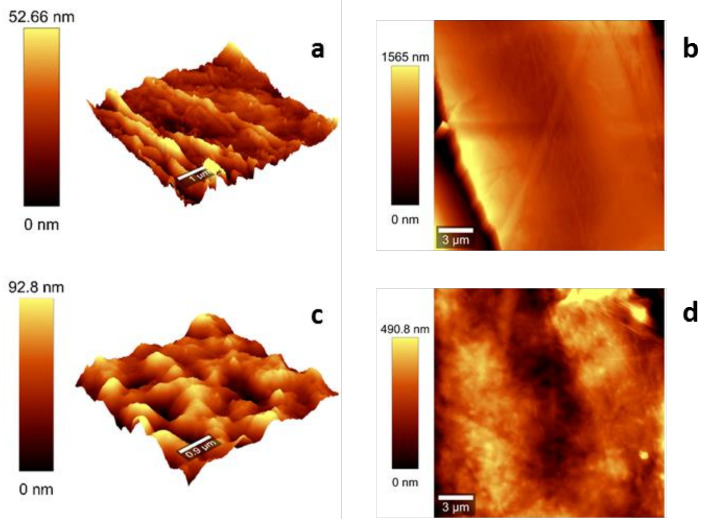
The 3D (**a**,**c**) and 2D (**b**,**d**) AFM surface topology images of uncoated (**a**,**b**) and (CL + LS) coated handsheet (**c**,**d**).

In addition to electrostatic interactions, the contribution of dispersion and donor–acceptor interactions of lignin with cellulosic fibers must be considered [16,32,33]. As discussed above, dispersion forces and hydrogen bonds may be particularly important for the interaction of negatively charged LS and cellulose fibers, whereas electrostatic forces may be predominant in the coating of HS with CL. Due to the large specific surface of cellulosic fibers and the easy penetration of aqueous solutions of lignin into the fiber networks, the effect of the fiber–lignin interaction must be much stronger than that of the interaction of lignin with the glass surface. This fact obviously should affect the structure of the coating layer. However, microscopy evaluation suggests that some similarities with regard to surface topology exist between paper and glass at the micron and submicron levels. This was concluded from the observation that CL + LS films showed largely similar surface patterns of alternating flat peaks and fringed depressions on both glass slides (Figure 1b) and the HS samples (Figure 5). As a result, the roughness of the coated fibers increased by ca. 50% compared to that of the uncoated HS samples (from ca. 10–15 nm to 15–20 nm). The observed similarity in surface and aggregate morphology encourages us to propose that the conclusions made about the structure of lignin films on the glass surface can be expanded to some extent for paper and cellulosic fiber networks, respectively.

**Figure 6:**
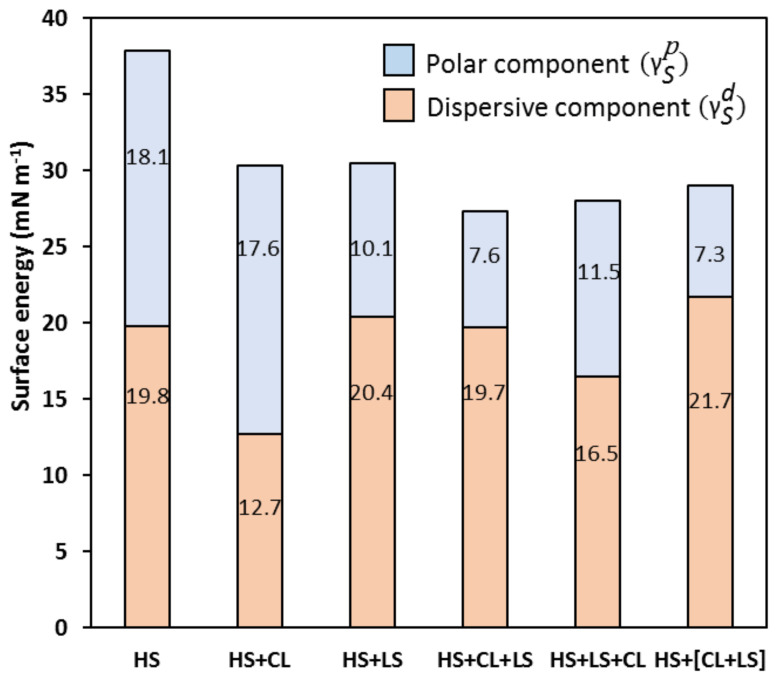
Calculated surface energy and its dispersive and polar components for lignin-free reference (HS) and single-layer coated handsheets (HS + CL; HS + LS) in comparison to two-step coating variants involving sequential deposition of cationic lignin and lignosulphonate with intermediate drying (HS + CL + LS); the reverse order of deposition with (HS + LS + CL) and without intermediate drying (HS + [CL + LS]).

This also includes the effects of lignin coating on water contact angle and moisture affinity. Similar to the coating experiments using glass lamina, the lowest WCA was observed when HS was coated with CL whereas the highest values were obtained for neat LS (Table 2) layers. CL + LS and LS + CL *all*-lignin coating resulted in intermediate WCA values, laying between that of LS or CL coating (Table 1). However, unlike the glass lamina coated with CL only (Table 1), HS coating with CL showed increased WCA when compared to the parent base paper (Table 2). Hence, despite the high hydrophilicity of CL, its interaction with the cellulose fiber imparts reduced water affinity to paper. This finding has been tentatively assigned to reduced total surface energy rather than to a substantial decrease in its polar component (Figure 6).

Calculation of surface energies and their dispersive and polar components revealed that, despite similarly high surface energy (ca. 30.3 mN m^−1^), the LS-coated HS specimen had an almost 61% higher percentage of this dispersive component when compared to CL-coated HS (Figure 6). This explains why much higher WCA values were measured for the HS + LS samples than for their HS + CL counterparts (Table 2). This fact also corroborates the previously suggested higher *C_PP_* values of the LS aggregates compared to that of CL which is supposedly due to the bulkier hydrophobic moieties of used lignosulphonate. The WCA values obtained for the CL + LS variant were close to that of LS coating. Application of the reverse coating order (variant LS + CL) resulted in lower WCA values, however, still being somewhat higher than for the CL coating. These findings are in accordance with the respective trends in the dispersive component of the total surface energies calculated for the different *all*-lignin coating variants (Figure 6).

Among all variants, [CL + LS] coating carried out without intermediate drying of the lignin layers featured the lowest polar and the highest dispersive component of the free surface energy (Figure 6). However, this did not translate into significantly higher WCA values compared to two-step CL + LS coating with intermediate layer drying (Table 2). This could be explained by practical difficulties in the coating of the moistened HS surface, resulting in overspreading and inhomogeneity of the coatings formed.

### 3.3. Surface Coatings on Printing Paper

**Figure 7:**
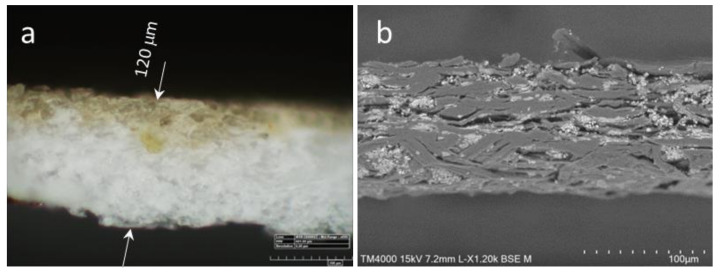
Cross-sectional optical micrograph of a handsheet (HS) coated by CL and LS (CL + LS) (**a**) and SEM image of the HS surface (**b**).

Aiming to study the effects of an *all*-lignin coating in a more practical context, various formulations tested before on glass slides and handsheets were repeated using an industrial prototype of uncoated printing paper produced from the same type of cellulosic fibers as HS (*E. globulus* bleached kraft pulp). This paper contained the sizing agent (ca. 1 wt.% of alkenyl succinic anhydride, ASA), inorganic filler (ca. 26 wt.% of CaCO_3_), and small amounts (<1 wt.%) of other common stock components (auxiliary resins, etc.). The paper was not surface-treated using any auxiliary formulation commonly used for finishing printing paper (e.g., starch/filler/adhesive formulations). Due to the higher porosity of this paper compared to HS, the penetration of aqueous lignin formulations in depth reached up to 30–40 µm (Figure 7a) whereas it was 10–30 µm for HS. In the paper assays, deposition of lignin appeared to occur almost exclusively at the surface of the cellulosic fibers only (no larger aggregates visible in the inter-fiber voids) whereas the inorganic filler is randomly distributed across the interstices at the surface and inside the bulk paper (Figure 7b). The deposition of CL and LS without drying intermediate layers (variant [CL + LS]) was not considered in these assays due to practical aspects.

**Figure 8:**
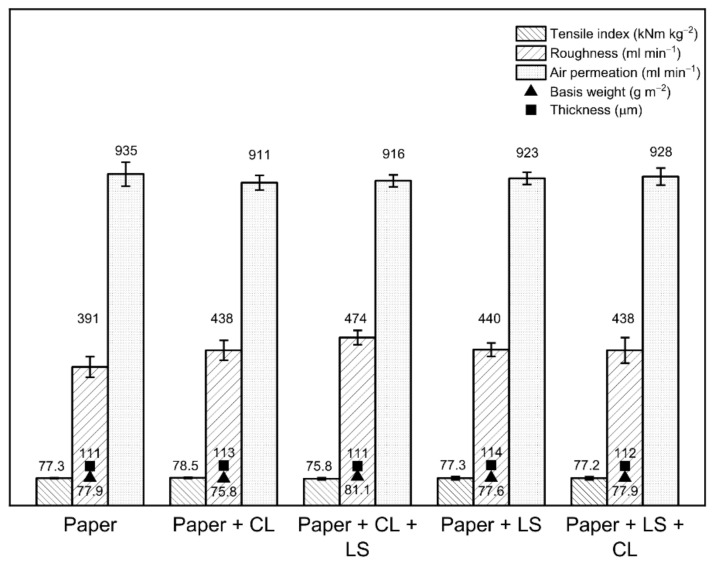
Tensile index, roughness, air permeability, basis weight, and thickness of the papers coated with: one layer of cationic lignin (CL); first layer of CL and second layer of lignosulfonates (CL + LS); one layer of LS and first layer of LS and second layer of CL.

Coating of the printing paper at the same application rate used for the handsheet series (5–6 g m^−2^) resulted in thinner coating layers due to the larger penetration depths. This entailed a less homogeneous distribution of lignin across the paper morphological elements (cellulosic fibers and filler particles) compared to the HS assay and made it more difficult to draw reliable conclusions with regard to the specific morphologies of the *all*-lignin structures formed upon coating which applies to both the level of conjugated structural elements and that of individual fibers (Figure S3, Supporting Materials). Nevertheless, an increase in paper roughness was observed after the deposition of the different lignin formulations (Figure 8). At the same time, the macroporous structure of the paper did not undergo major changes no matter which type of lignin formulation had been applied. This was concluded from largely similar air permeability values of uncoated and coated papers (Figure 8). In addition, the physical properties (e.g., tensile index) also did not show some significant changes.

**Figure 9:**
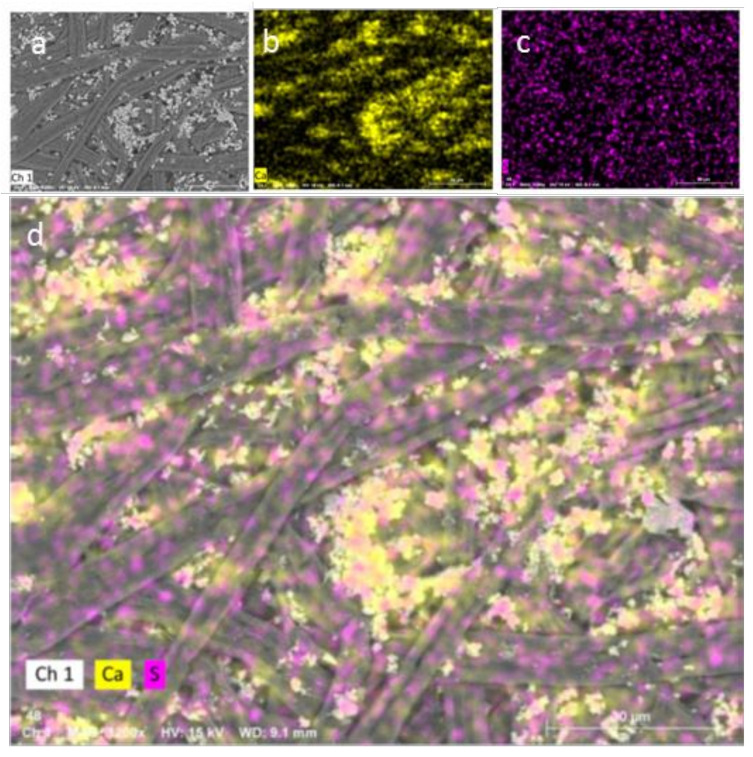
SEM (**a**) and SEM/EDS images (**b**–**d**) of the paper surface coated with LS. The mapping images show the distribution of Ca from CaCO_3_ ((**b**), yellow color), S from LS ((**c**), violet color) and the combined image of Ca and S distribution (**d**).

The much lower affinity of industrial paper for water (compared to HS, Table 2 and Table 3) caused by the presence of the sizing agent (ASA) was found to affect all properties of the experimental papers coated with the different lignin formulations. However, regardless of the lower water affinity of the source material (WCA 86°), *all*-lignin coated papers were even more hydrophobic (WCA between 89° and 115°, Table 3). For the different types of lignin formulations, similar tendencies were found with regard to the effects on WCA as in the coating of HS (Table 2) though possessing higher WCA. Among the tested variants, single CL followed by dual LS + CL coating had the least effect with regard to enhanced hydrophobicity whereas the gain in WCA was already remarkable for the reverse dual coating CL + LS (85.9° vs. 99.9°, Table 3) and highest for single LS coating. As discussed before, these results are, at least partially, due to the different affinity of CL and LS to the major paper components, i.e., cellulosic fibers and inorganic fillers. Correspondingly, the SEM/EDS analysis of the paper coated with LS clearly showed almost isotropic coverage of the surface (Figure 9) which includes both cellulosic fibers and CaCO_3_ particle aggregates.

**Figure 10:**
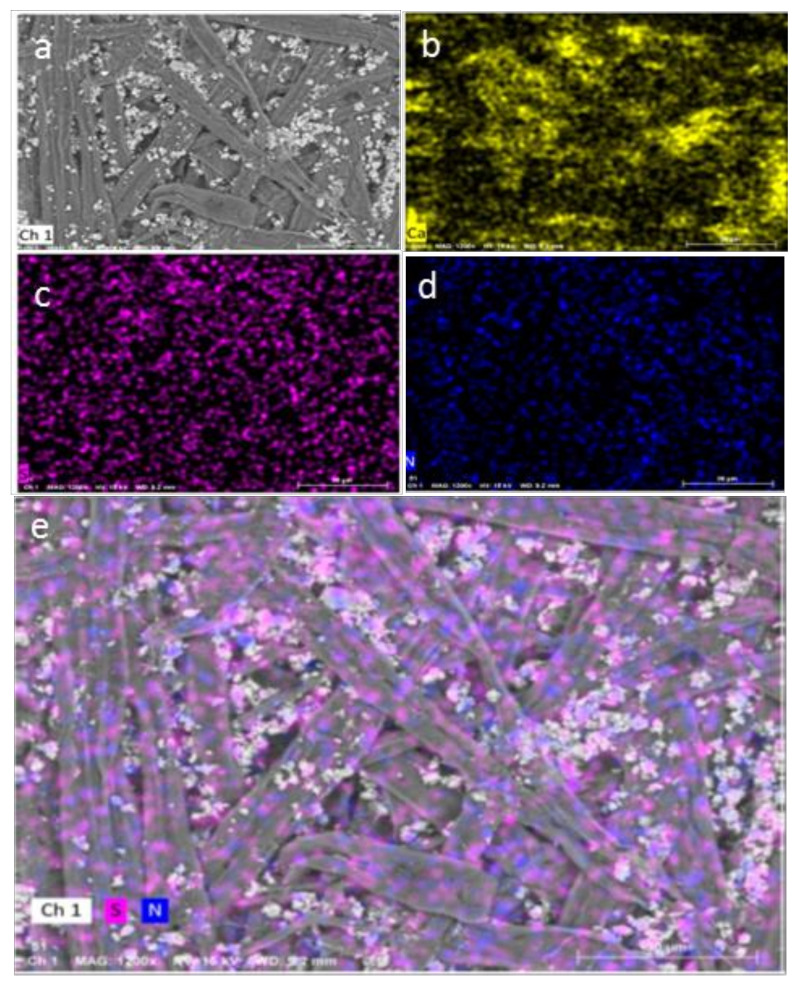
SEM (**a**) and SEM/EDS images (**b**–**e**) of industrial printing paper dual-step surface coated with LS and CL (LS + CL). The mapping images show the distribution of Ca from CaCO_3_ ((**b**), yellow color), S from LS ((**c**), violet color), N from CL ((**d**), blue color) and the combined image of S and N distribution (**e**).

This even and isotropic distribution of LS is supposedly caused by both electrostatic attraction of positively charged CaCO_3_ particles and negatively charged LS and by chemisorption of LS on the cellulosic surfaces. Furthermore, the deposition of poorly soluble calcium salts of lignosulfonates can occur, promoted by the slightly acidic pH of the LS coating solution (pH ca. 5) inducing partial dissolution of the carbonate particles and engaging calcium ions to form LS calcium salts. This was not the case for the dual-step LS + CL coating. When CL was deposited on the LS pre-coated paper, preferential coating of the cellulose fibers took place (Figure 10). The CaCO_3_ particle aggregates were coated only in the regions with previously deposited LS. This is explained by the electrostatic repulsion of positively charged CaCO_3_ and CL. Thus, some paper surface regions with exposed inorganic fillers and surrounding areas were not covered effectively by CL which led to the aforementioned peculiarities of paper surface wetting with water.

Except for the single-step CL coating, all other lignin formulations tested for surface modification of the industrial printing paper were capable to impart enhanced hydrophobicity (*θ* > 90°). As with the coatings on the glass lamina and HS samples (Table 1 and Table 2), deposition of solely LS on the paper gave the highest WCA (ca. 115°), followed by consecutive CL and LS coating (CL + LS, Table 3). This coherence in the wetting behavior of either glass, laboratory handsheets, or industrial printing paper with different lignin formulations allows the proposition of similarity in lignin deposition mode on these substrates though, obviously, the characteristics of the different substrates in terms of topologies and compositions interfere to a certain extent. The evaluation of potential lignin leaching from the coated industrial papers (water drop proof) confirmed the high stability and negligible leaching for coating variant CL + LS whereas noticeable coating erosion occurred when using CL alone.

## 4. Conclusions

The results of this study demonstrated for the first time the possibility of coating cellulosic substrates with *all*-lignin formulations in a layer-by-layer deposition approach using the combination of quaternized kraft lignin (CL) and lignosulphonate (LS). The electrostatic attraction between positively charged CL and negatively charged LS leads to the formation of insoluble self-organized macromolecule aggregates, whose structure depends on the order and the mode of their application on the paper surface. It was proposed that the structure of those aggregates can be represented as the result of the interaction of amphiphilic water-soluble lignin molecules leading to their ordered mutual arrangement. These structural aggregates are able to interact with each other to form films on the cellulosic fibers of the paper, providing lower free surface energy, mainly governed by its polar components, thus promoting hydrophobic properties. Although continuous layer coating by *all*-lignin formulations was impossible for industrial paper due to its inherent highly macroporous structure, fiber-level coating allowed for a significant decrease in air permeability without deteriorating the paper’s strength properties. Coating uniformity of the paper with *all*-lignin formulations can be affected by different affinities of the paper ingredients, such as inorganic fillers, with lignin formulation components promoting synergetic effects. It can be inferred, by analogy with literature data, that coating with *all*-lignin formulations will change the permeability, printability, antioxidant, and antimicrobial/antifungal properties of paper, among others. Furthermore, the application of *all*-lignin formulations to pre-coated papers can overcome the obstacles of inhomogeneous coating and impart new interesting properties to these materials. This work is in progress.

## Figures and Tables

**Table 1:** Contact angles with water of the films deposited on the glass lamina.

* Coating Formulation	Deposition Mode	Contact Angle (°)
(GL)	-	52.1 ± 1.0
CL	Drying of the deposited layer	32.5 ± 1.8
LS	Drying of the deposited layer	68.4 ± 2.1
CL + LS	Drying of the intermediate layer	61.7 ± 3.1
LS + CL	Drying of the intermediate layer	41.3 ± 1.0
[CL + LS]	No drying of the intermediate layer	64.7 ± 2.8

* CL—cationic lignin; LS—lignosulphonate; GL—glass lamina.

**Table 2:** Water contact angles of handsheets subjected to different lignin coating assembles.

* Coating Formulation	Deposition Mode	Contact Angle (°)
(HS)	-	63.0 ± 1.5
CL	Drying of the deposited layer	72.7 ± 2.6
LS	Drying of the deposited layer	86.4 ± 2.1
CL + LS	Drying of the intermediate layer	81.9 ± 1.2
LS + CL	Drying of the intermediate layer	75.5 ± 2.0
[CL + LS]	No drying of intermediate layer	82.0 ± 1.8

* CL—cationic lignin; LS—lignosulphonate.

**Table 3:** Water contact angles of industrial printing paper coated with lignin formulations.

* Coating Formulation	Deposition Mode	Contact Angle (°)
(paper)	-	85.9 ± 2.6
CL	Drying of the deposited layer	89.3 ± 2.8
LS	Drying of the deposited layer	114.8 ± 2.1
CL + LS	Drying of the intermediate layer	99.9 ± 3.3
LS + CL	Drying of the intermediate layer	94.0 ± 3.1

* CL—cationic lignin; LS—lignosulphonate.

## Data Availability

Data sharing not applicable.

## Supplementary Materials

The following are available online at https://www.mdpi.com/article/10.3390/ma15227869/s1, Figure S1: Mixtures (*v*/*v*) of 1 wt.% solutions of cationic eucalyptus lignin (CL) and eucalyptus lignosulphonates (LS); Figure S2: 3D AFM surface topology image of the glass lamina coated with CL.; Figure S3: 3D (**a**,**b**) and 2D (**c**,**d**) AFM surface topology images of the uncoated (**a**,**c**) and (CL + LS) coated (**b**,**d**) industrial paper.

## Abbreviations

SEM	Scanning electron microscopy
AFM	Atomic force microscopy
SEM/EDS	Scanning electron microscopy coupled with energy dispersive spectroscopy
