# Supplementary material for: Modification of Paper Surface by All-Lignin Coating Formulations
Source: Materials (Basel). 2022 Nov 8;15(22):7869. doi: 10.3390/ma15227869 (PMC9695548; doi:10.3390/ma15227869)
Supplement: Supplementary file 1 [file materials-15-07869-s001.zip › materials-1990908-supplementary.pdf]

Supplementary Materials

Modification of paper surface by *all*-lignin coating formulations

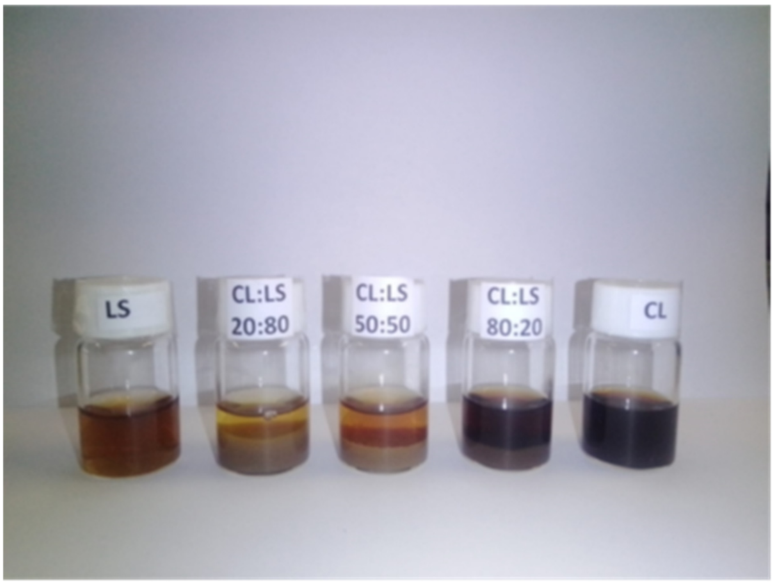

Figure S1. Mixtures (v/v) of 1 wt.% solutions of cationic eucalyptus lignin (CL) and eucalyptus lignosulphonates (LS).

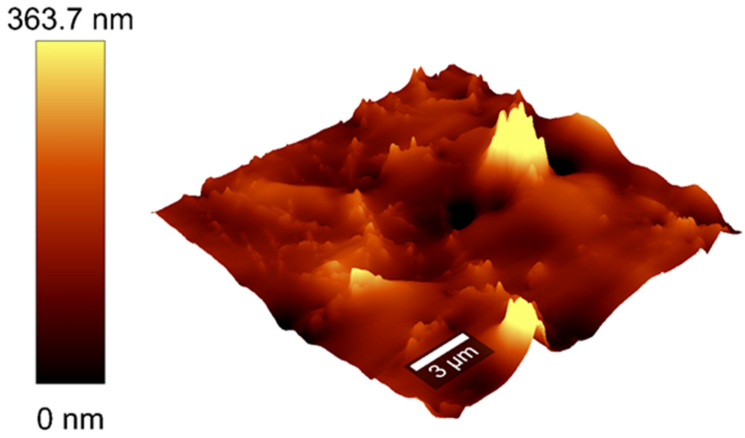

Figure S2. 3D AFM surface topology image of the glass lamina coated with CL. The size of image is of 20 x 20 µm.

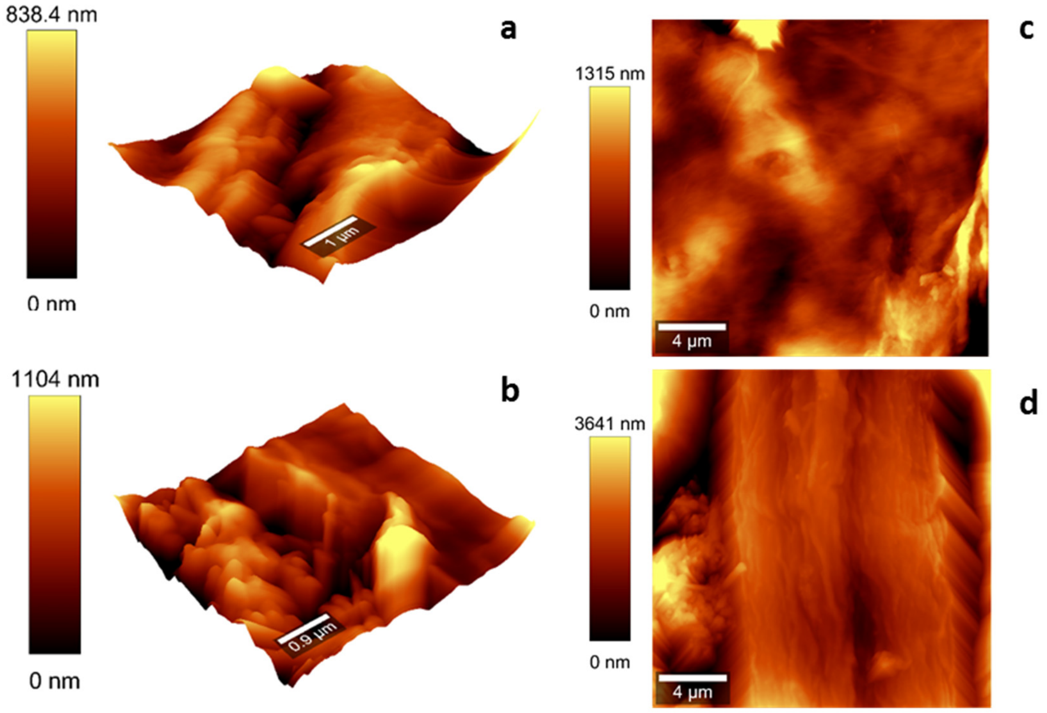

Figure S3. 3D (a, b) and 2D (c, d) AFM surface topology images of the uncoated (a, c) and (CL +LS) coated (b, d) industrial paper.
